# Elucidation of Nuclear and Organellar Genomes of *Gossypium hirsutum*: Furthering Studies of Species Evolution and Applications for Crop Improvement

**DOI:** 10.3390/biology2041224

**Published:** 2013-10-18

**Authors:** Jocelyn A. Moore, Caryl A. Chlan

**Affiliations:** Biology Department, the University of Louisiana at Lafayette, Lafayette, LA 70504, USA; E-Mail: jam8941@louisiana.edu

**Keywords:** cotton, polyploidy, fiber, gossypol, plastid

## Abstract

Plant genomes are larger and more complex than other eukaryotic organisms, due to small and large duplication events, recombination and subsequent reorganization of the genetic material. Commercially important cotton is the result of a polyploidization event between Old and New World cottons that occurred over one million years ago. Allotetraploid cotton has properties that are dramatically different from its progenitors—most notably, the presence of long, spinnable fibers. Recently, the complete genome of a New World cotton ancestral species, *Gossypium raimondii*, was completed. Future genome sequencing efforts are focusing on an Old World progenitor, *G. arboreum*. This sequence information will enable us to gain insights into the evolution of the cotton genome that may be used to understand the evolution of other plant species. The chloroplast genomes of multiple cotton species and races have been determined. This information has also been used to gain insight into the evolutionary history of cotton. Analysis of the database of nuclear and organellar sequences will facilitate the identification of potential genes of interest and subsequent development of strategies for improving cotton.

## 1. Introduction

Cotton has been cultivated by man for thousands of years. Because cotton fiber has desirable properties for the production of textiles, cotton has been the subject of intense cultivation and breeding over the centuries. These efforts have now expanded to molecular analysis of the cotton genome and studies to determine the extent that specific genes or groups of genes contribute to the desirable properties of cotton. Although cotton is mainly valued for its fiber, cotton by-products are used in human food and animal feed. Genomics studies of cotton can be applied to the improvement of the nutritional quality of cottonseed products.

The genus *Gossypium* is diverse and has both diploid and tetraploid species [1]. There are 45 diploid species and five tetraploid species of cotton. The diploid species have been placed into genome types labeled A through G and K. Four of these diploid species comprise the African group: A, B, E and F [2,3,4,5]. Three diploid species are found in Australia (C, G and K), and the D species is native to the New World. Most of the cotton fiber produced today is a product of lint harvested from four species: two Old World diploid lines (*G. herbaceum* L. and *G. arboreum* L.) and two tetraploid lines (*G. hirsutum* L. and *G. barbadense* L.). Approximately 90% of the global cotton production is from *Gossypium hirsutum*, a tetraploid species [6].

Polyploidy is common in flowering plants and provides a potential mechanism for the development of new genetic characteristics. Cotton is an excellent model system to study the effects of polyploidy and subsequent gene expression on phenotype through the introduction of new genetic traits. Between one and two million years ago, tetraploid cotton species arose in the New World as the result of hybridization between an A genome diploid from the Old World (ancestral African/Asian species) and a D genome diploid from the Americas [7]. The closest existing relatives of the ancestral genome parental species are the A diploids, *Gossypium herbaceum* and *Gossypium arboreum*, and the D diploid, *Gossypium raimondii*. Formation of the tetraploids resulted in enhanced fiber length and mass; however, intensive breeding for desirable fiber properties has resulted in decreased genetic diversity and the loss of potentially desirable characteristics, such as resistance to pests or tolerance to drought. Genomic analysis of the cotton progenitor strains is currently under way, and the complete sequence of *G. raimondii* has recently been published [8,9]. This information will lead to additional insights as to the fate of duplicated plant genomes.

The three major cotton producing countries are China, India and the United States. The economic impact of the cotton industry is immense—in the United States alone, the 2012 cotton crop was valued at ~6 billion dollars for the fiber and ~1.5 billion dollars for cottonseed oil [10]. Cotton is expensive to grow: conventionally grown cotton requires regular pesticide and fertilizer applications that cannot only be expensive, but can be detrimental to the environment and animal populations. In recent years, efforts have been made to improve the yield of cotton while, at the same time, decreasing the growing costs and environmental impact.

Genetically modified (GM) cotton that expresses anti-insect and herbicide-resistant genes (Bt and RoundUp Ready cotton) is now widely grown. However, concerns about the lack of variability in the germplasm and the potential for the development of resistance to the engineered traits has led to several new strategies to identify natural resistance traits in germplasm stocks, as well as development of transformation strategies that result in crops that are more universally accepted.

A timeline showing some of the key advances in the analysis of cotton genes and gene products is presented in Table 1. Studies have included isozyme comparisons in the 1980s [11], analysis of restriction fragment length polymorphisms (RFLPs) in the 1990s [7,12], microsatellite analyses in the 2000s [13,14] and whole genome sequencing of *G. raimondii* (a D genome ancestral diploid cotton) in 2012 [8,15]. Genomic analysis and mapping studies will enable researchers to identify native genes or combinations of genes that confer desirable properties, such as enhanced resistance to pathogens or drought, improved fiber quality and improved nutritional composition of cottonseed. Genomic information will also advance the establishment of transformation approaches that result in recombinant plants containing genes identified and isolated from cotton germplasm with known sites of transgene integration. 

**Table 1:** Partial timeline for genetic studies of *Gossypium*. RFLP, restriction fragment length polymorphism. EST, expressed sequence tag.

Date	Approach	Cultivar (s)/Tissues	Reference
1989	Isozymes	*G. arboreum*	[11]
		*G. herbaceum*	
1994	RFLP	*G. hirsutum*	[12]
		*G. barbadense*	
1999	RFLP	*G. herbaceum* L. × *G. arboreum* L.	[7]
		*G. trilobum* Skovsted × *G. raimondii*	
		*G. hirsutum* “palmeri” × *G. barbadense*	
2003	Microsatellites	*G. hirsutum*	[13]
		*G. arboreum*	
2007	Microsatellites	*G. arboreum*	[14]
		*G. herbaceum*	
		*G. thurberi*	
		*G. raimondii*	
		*G. gossypioides*	
		*G. hirsutum*	
		*G. barbadense*	
2010	Physical Map	*G. raimondii*	[15]
2012	EST	*G. arboreum* (gynoecium, calyx, fiber, roots, whole seedlings)	[16]
		*G. arboreum* (cotton fiber 7–10 days post anthesis (dpa))	
		*G. raimondii* (gynoecium, calyx, fiber, roots, whole seedlings)	
		*G. raimondii* (meristem, calyx, fiber, root, petal, seedling)	
		*G. raimondii* (whole seedling, normalized floral organs including developing embryos)	
		*G. hirsutum* (gynoecium, calyx, fiber, roots, whole seedlings)	
		*G. hirsutum* (bud, leaf, stem, whole seedling (with roots))	
		*G. hirsutum* (ESTs that are publically available in GenBank)	
		*G. barbadense* (bud, leaf, stem, whole seedling (with roots))	
		*G. barbadense* (bud, leaf, stem, whole seedling (with roots))	
		*G. barbadense* (ESTs that are publically available in GenBank)	
2012	Genome Sequencing	*G. raimondii*	[8]
2012	Genome Sequencing	*G. raimondii*	[9]

## 2. Genome Insights into Polyploidy and Evolution of Cotton

Many angiosperms exhibit polyploidy, due to one or multiple gene duplication events that have occurred throughout evolution. Analysis of chromosomal numbers results in estimates that polyploidy occurred in the history of 56% to 70% of flowering plants [17,18,19]. Genomic studies [20] support the possibility that duplication events may have preceded angiosperm evolution, which would mean that all flowering plants have experienced some degree of polyploidy in their evolutionary history. Gene duplication events can lead to several outcomes: maintenance of original function, subfunctionalization, formation of pseudogenes or development of novel functions [21]. Subfunctionalization suggests that duplicated gene expression may be partitioned [21].

Cotton has proven to be an excellent model species to study polyploidization, because commercially cultivated cotton, *Gossypium hirsutum*, is allotetraploid [21]. In allotetraploid cotton, nuclear genes were simultaneously duplicated from an Old World ancestor (A genome) and a New World ancestor (D genome). Old World cotton is native to Asia and Africa and is now represented by *Gossypium arboreum* and *Gossypium herbaceum*. Old World cotton produces seeds with spinnable fibers, which aid in seed dispersal near aquatic habitats. In contrast, New World cottonseeds (such as those from *G. raimondii*) have no spinnable fibers. Five tetraploid species of cotton are recognized: *Gossypium hirsutum*, *Gossypium darwinii*, *Gossypium barbadense*, *Gossypium tomentosum* and *Gossypium mustelinum*. *G. barbadense* and *G. hirsutum* are the main commercially cultivated cottons. *G. barbadense* is desirable for its excellent fiber properties (long, fine, strong) and includes Pima, Egyptian and Sea Island cotton [5].

To gain insight into the fate of duplicated genes in cotton, the expression patterns of quantitative trait loci (QTLs) associated with fiber length, thickness and maturity within A or D subgenomes of *G. hirsutum* and *G. barbadense* were identified. Of the 14 QTLs that were found to be associated with fiber traits, 10 mapped to linkage groups in the D subgenome [22]. This clearly indicates a D subgenome bias with respect to fiber properties [21]. This bias cannot be explained by large differences in genetic variation between the A and D genomes. Restriction fragment length polymorphism studies showed that 42% of the markers mapped to the A genome, while 46% mapped to the D genome. The bias of fiber property markers for the D genome is intriguing, because the D genome cottons do not produce fibers. It has been suggested that the D genome cottons were not subjected to the same selective pressures as the A genome cottons. This may be due to both natural selection for improved fiber formation associated with seed dispersal and subsequent human breeding for more fiber in A genome cottons [23]. It was only after the formation of polyploidy cotton (fiber producing) that the effects of the D genome on fiber properties could be observed. If this is accurate, then the QTLs associated with cotton fiber properties in the D genome may help locate sites in the A genome that have been targets for selection and trait fixation [22]. 

Since 1998, more studies identifying QTLs associated with fiber properties have confirmed a D genome bias [24,25]. As many as 112 identified QTLs related to fiber properties are present in the D subgenome of allopolyploids, while the A subgenome has roughly 84 [25]. Some clustering of QTLs in certain regions of multiple chromosomes within the allopolyploid genome was observed. Chromosome 17 of the D subgenome contained the most QTLs [25]. QTL analysis over the years suggests that the evolution of D subgenome fiber related alleles is occurring more quickly than that of the A subgenome, thus leading to a D genome bias [25]. D genome bias was also observed in microarray analyses of all five allopolyploids and a synthetic diploid F1 hybrid [26]. Comparisons of gene expression in fiber, floral and vegetative tissue showed variation in the extent of genome bias between tissue types [27]. 

## 3. Cotton Improvement—Simple and Complex Traits

Modification of cotton properties, either by conventional breeding, genetic engineering or a mixture of the two strategies is an ongoing process driven by the desire to improve yield, quality and the net value of the crop. Cotton is valued for both its fiber and cottonseed. Cottonseed by-products include cottonseed oil (used in many food products) and cottonseed meal (used as animal feed). Biotechnology has enabled plant biologists to introduce new genes and traits within and between species. Since GM crops were first introduced in 1996, the number of hectares planted with GM crops has increased by 100-fold—from 1.7 million hectares to more than 170 million hectares in 2012 [28]. Biotech cotton was grown on 24.3 million acres in 15 different countries and constituted 81% of the cotton crop [28]. The traits expressed included insect resistance, herbicide resistance and combined resistance to insects and herbicides. The steps involved in the generation of these transgenic cotton lines, as well as the identification of some additional targets for cotton improvement are discussed below.

Pathogen and Herbicide Resistance: Initially, cotton characteristics targeted for biotechnological modification were associated with improved yield and crop value. The first commercially available GM cotton was released in 1996 and expressed a *Bacillus thuringiensis* (Bt) toxin gene. The δ endotoxins, naturally produced by some strains of *Bacillus thuringiensis*, accumulate in crystalline bodies within the bacteria. Different strains of Bt produce different Cry proteins, with differing specificities and activities [29,30,31]. Initially, four groups of Cry proteins (Cry I to Cry IV) were identified based on the Cry protein activity against Lepidoptera, Diptera, Coleoptera or nematodes [32]. Currently, over 100 Cry proteins are recognized based on a classification scheme described by Crickmore [33].

The Bt toxin (Cry protein) has been used for years as a compound to control cotton pests. Once ingested, the crystals of Cry proteins are released, and the Cry proteins are digested by insect proteases. This results in the generation of a trypsin-resistant, active endotoxin core that binds to brush border cell receptors. Binding facilitates the formation of pores, which change the membrane permeability, ultimately resulting in cell lysis [31,34]. The activity of the toxin depends on the efficiency of toxin release from the crystalline structures (pH-dependent), cell receptor binding specificity and the binding constant, as well as the number of receptors.

Bollgard^TM^ cotton (Monsanto, Saint Louis, MO, USA) was the first commercially produced cotton. It was engineered to express a modified CryIA protein. Initial experiments constitutively expressing the wild-type CryIA protein were disappointing. The levels of transgene expression were low—less than 0.001% of the total leaf soluble protein [35]. The CryIA gene was modified in efforts to improve expression levels. These modifications included introduction of only the active toxin region (a truncation of the wild type protein), codon utilization optimization for plant expression and removal of sequences that could result in the instability of the mRNA [36]. The modified CryIA protein (CryI(c)) was expressed at higher levels in transgenic cotton leaves and shown to be active against two major cotton pests—the boll worm (BW) and tobacco bud worm (TBW) [36]. Additional greenhouse and field trials clearly demonstrated that Bt cotton was resistant to BW and TBW: mortalities close to 100% were observed 1–4 days after insects had fed on transgenic cotton [37,38]. In addition, pink boll worm damage was reduced by up to 95% [39,40,41]. Cabbage loopers and other leaf perforators are also susceptible to Bt toxin [41,42]. 

After the introduction of the first GM cotton, additional traits, such as herbicide resistance, have become available, either as single modifications (RoundUp Ready) or stacked combinations (RoundUp Ready/Bt). The use of GM cotton has expanded world-wide and is common in all the major cotton producing countries. In 2012, transgenic cotton was grown in 15 countries (both industrialized and developing), and it was estimated that 81% of the global cotton planted was transgenic [43].

Additional efforts to improve growing conditions and the cost effectiveness of cotton are under investigation. In addition to insect pests, cotton is subject to other biotic stresses, including a variety of fungal diseases, such as wilt (*Fusarium* wilt, *Verticillium* wilt) [44] and boll rot (many etiological agents, including *Aspergillus flavus*). *A. flavus* is particularly troublesome, as many strains of *A. flavus* can produce aflatoxin, a carcinogenic compound. Aflatoxin levels in cottonseed dramatically impact the seed value, and cottonseed income can account for between 10% and 15% of the value of a cotton crop [45]. When the cottonseed aflatoxin levels exceed mandated levels (20 ppm for food and animal feed in the United States) [46], either the seed must be further processed (an additional expense) or, in some cases, deemed unusable. A variety of strategies are being pursued to reduce aflatoxin levels in cotton, including seeding cotton fields with atoxigenic strains [47,48] and genetic engineering [49,50]. Genetic engineering efforts have included the expression of potential anti-fungal genes or peptides singly or in combination to inhibit fungal growth.

New advances in the genomics of cotton, such as those performed in peanut [51], may be helpful in the identification of genes in cotton germplasm that confer enhanced resistance to fungal pathogens. To date, high levels of natural resistance to *A. flavus* infection has not been reported for commercially available cotton cultivars, but analysis of ancestral populations may result in the identification of genes of interest that have been lost over the years of breeding current cotton varieties.

Abiotic Stress: As water becomes more and more limiting, there is added pressure to grow crops with less irrigation. Abiotic stress associated with salinity, temperature extremes and fluctuation and accumulation of toxic compounds will increase in response to global climate change and responses to increased demand for higher and higher yield to meet demand [52,53]. Development of drought tolerant cotton has long been a target for genetic engineering, but it is a difficult undertaking. Strategies to confer or enhance natural drought tolerance in cotton have recently been reviewed [54]. Efforts to identify the genetic interactions involved in cotton response to water stress have utilized a variety of approaches. These include QTL mapping studies, such as those described by Saranga *et al*. [55], in which the progeny of crosses between *G. hirsutum* and *G. barbadense* were analyzed to determine which physiological factors changed in concert under water stress and the effect of those changes on overall productivity. Marker-assisted selection (MAS) has been used to study the exchange of QTLs associated with drought tolerance and their effect on productivity [56]. The consensus of these studies was that conventional breeding in addition to molecular-based approaches will be necessary to generate plants with enhanced drought tolerance. Other studies focused on drought-induced differential gene expression in cDNA libraries [57].

Transcriptomics studies have identified cotton genes that are expressed in response to water stress [58,59]. Based on the results of a study of *G. hirsutum* (tetraploid cotton), it is clear that response to water stress is a complex process that results in large numbers of upregulated and down-regulated genes [60]. Studies compared osmotic stress expression profiles in four different genotypes of *G. herbaceum*, a diploid cotton (A genome) that is preferentially grown in arid regions [61]. Fourteen accessions of *G. herbaceum* were screened to identify the most tolerant and most resistant members, and these accessions were chosen for further study [62]. Leaf gene profiles were compared between these accessions after they were subjected to mild and moderate water stress. The drought resistance accession Vagard differed from the drought sensitive RAHS-14 in many aspects associated with biological processes, cellular components and molecular function (including differences in levels of expression of specific transcription factors). The resistant Vagard exhibited significantly higher levels of gene ontology (GO) termed “responses to abiotic stresses”, and the sensitive RAHS-14 expressed a unique set of transcription factors associated with senescence. Transcription profile comparisons of roots from two drought tolerant and two resistant *G. herbaceum* accessions (Vagard, GujCot-21, RAHS-IPS-187 and RAHS-14) showed that drought tolerant lines expressed higher levels of antioxidant associated transcripts (based on GO), expressed cell wall synthesis transcripts at significantly lower levels than the sensitive lines and exhibited different expression profiles of membrane transporters [59]. 

Fiber: Cotton fibers are elongated trichomes that exhibit properties desirable for the production of textiles. Fiber properties, such as tensile strength, length and fineness, are associated with the value of cotton as a fiber crop. Cotton fibers can vary in length from 2–3 cm in *G. hirsutum* (the most commonly grown cotton cultivar) to over 6 cm in *G. barbadense* (considered an extra-long staple cotton). In contrast, the diameter of a cotton fiber is relatively thin: *G. hirsutum* fibers have an average diameter of 11–22 µm [62]. The niche that cotton currently holds could be expanded if improvements can be made in other categories, such as flame resistance and resiliency [34,63]. The progress and challenges associated with improving cotton fiber have been previously reviewed [64,65]. Most recently, genetic mapping and transcriptomics studies have begun to shed light on the processes and genes that interact to generate desirable properties in cotton fibers. In 2004, Arpat *et al*. published a study of expressed sequence tags (ESTs) associated with different stages of fiber development in *G. arboreum*, a diploid cotton [66]. Surprisingly, the fiber transcriptome represents a large portion of the cotton genome—at least 35%–40%. Formation of cotton fibers has been divided into four stages: initiation, elongation, formation of the secondary cell wall and fiber maturation [62]. Microarray studies demonstrated that dynamic changes occur between and during these stages, with expansion-associated gene expression high during primary cell wall synthesis, and the secondary cell wall synthesis stage was characterized by increased expression of transcripts associated with the formation of secondary cell walls and metabolism [66]. To gain further insight into the changes in gene expression that are associated with desirable cotton characteristics, additional studies have focused on differential gene expression in the fiber of wild *versus* domesticated cotton accessions of *G. barbadense* [67], differential expression in fibers of *G. hirsutum* *versus* *G. barbadense* [68,69,70], as well as differences in transcript or protein expression between fiber mutants and wild-type [71,72,73,74].

Because *G. barbadense* produces high yields of superior quality cotton, it has been the subject of many studies to determine how gene expression in the fiber of this species differs from that of other closely related species. The fibers of the more widely planted, commercially important *G. hirsutum* are not as long or silky as those of *G. barbadense*. During the process of domestication, plants were selected for a shorter life cycle (annual as opposed to perennial) and improved fiber quality and length [67]. One study compared expression profiles of fibers isolated from early, mid and late elongation stages of a wild and a domesticated accession of *G. barbadense*. This study corroborated the complexity of the *G. barbadense* genome and demonstrated that the expression of many genes changed during development in both domesticated and wild accessions. Greater changes in gene expression were observed at different developmental stages within an accession than were observed between accessions. Based on the results, the authors proposed a model for fiber development [67]. Subsequent studies of a *G. barbadense* developing fiber cDNA library resulted in the generation of 10,979 ESTs that have been deposited into a public database. These data will assist studies designed to understand fiber development processes and the development of breeding programs [75].

Transcriptional profiling, genetic mapping and deep sequencing have been used to identify differences between genes involved in fiber development and formation in *G. barbadense* *versus* *G. hirsutum*. In these studies, genes associated with secondary metabolism and pectin synthesis and modification varied the most between the species, and most of the differences occurred before the fiber begins to thicken [68]. Deep sequencing of ESTs from *G. hirsutum* and *G. barbadense* at two different stages of fiber development again revealed a complex set of interactions and processes involved in the different stages of fiber development [69]. Approximately 70% of the transcripts that exhibited differential gene expression were upregulated at 10 dpa compared to expression levels at 22 dpa. This difference was greater in *G. hirsutum* compared to *G. barbadense* and may reflect a difference in the time course of fiber formation between the two species. This hypothesis is supported by the fact that *G. barbadense* has more overexpressed genes at 22 dpa than *G. hirsutum* [69]. These studies contributed to the library of cataloged genes and markers that are associated with fiber development. A recent study has expanded the suite of molecular markers associated with fiber development [70]. Using single-strand conformation polymorphism (SSCP) studies, the authors were able to identify 37 gene and 21 protein polymorphisms [70]. Because fiber formation is such a complex process, it was not surprising that the authors found many genes were present in multiple GO categories. It was not until a level 3 analysis that the differences between the proportion of genes in different categories was observed: hydrolases accounted for 24% of the molecular function category, and biosynthetic processes accounted for the majority (33%) of molecular functions [70]. 

Efforts to identify specific genes, products and interactions associated with complex fiber traits have also focused on studies of cotton fiber mutants, such as fuzzless or lintless lines. As a result of the studies discussed above, as well as many other transcriptomic, proteomic and genomic analyses, we now have a much more complete map of the cotton genome and greater representation of markers for *G. barbadense*. Future determination of the genome sequence of an ancestral Old World cotton progenitor in conjunction with the recently described genome of the New World representative, *G. raimondii*, will enable researchers to identify the origins of genes that contribute to commercially desirable fiber properties.

Gossypol: Gossypol, a phenolic compound, is found in stems, leaves and roots of *Gossypium* species [76]. It accumulates in the pigment glands of cotton and acts as a chemical deterrent to pathogen infection [77,78]. Unfortunately, free gossypol is toxic to most mammals, which limits the versatility of cottonseed meal for use in food and feed. Ruminants can ingest more cottonseed meal containing gossypol than monogastric animals, because the free gossypol is detoxified in the rumen [79]. Currently, cottonseed meal is mainly used to augment animal feed as a substitute, at least in part, for soybean meal.

In addition to being toxic, free gossypol can bind with lysine residues, which reduces the nutritional value of the meal [80]. Reduction of gossypol content in cotton to develop cottonseed and cotton meal with the potential to feed a wide spectrum of animals, including humans, has long been a goal for cotton breeders. In the 1950s, a glandless cotton mutant was described [81,82]. World-wide efforts ensued to establish this trait in commercially acceptable varieties. The nutritional content of cottonseed meal derived from glandless cotton was acceptable; however, glandless cotton was prone to insect damage in the field [82]. More recently, a genetic engineering strategy has been implemented to reduce gossypol levels specifically in cottonseed. A cottonseed storage protein promoter [83] was used to generate an RNAi construct to silence the expression of a gene family that encodes (+)-delta-cadinene synthase [84]. Transgenic cotton that harbored the seed-specific promoter/(+)-delta-cadinene synthase RNAi construct exhibited reduced seed gossypol levels (~98% reduction in T2 seeds). Reduction in gossypol was restricted to the seed, and this trait was stable throughout multiple generations [85]. Additional insights into the cotton genome may be used to develop modified strategies to further reduce gossypol levels in cottonseed or to improve the nutritional content. Production of cottonseed that is acceptable for human consumption could conceivably produce enough protein to feed half a billion people annually [86].

## 4. Development of New Transformation Strategies

Nuclear: *Agrobacterium*-mediated transformation and biolistic transformation are the two most commonly used methods to engineer plant cells. Although these methods are broadly applicable to a wide variety of plant species, there are disadvantages associated with both of these approaches. Multiple copies of the transgene may be incorporated into the recipient genome, which can trigger genetic silencing. Gene silencing can occur transcriptionally or post-transcriptionally in plants, and the probability of its occurrence is related to the number of gene copies present [87]. The gene of interest may also not be expressed, because it integrated into a “silent” site in the genome (e.g., heterochromatin). The gene of interest may affect the expression of other genes: insertion of the transfer DNA (T-DNA) can be accompanied by small deletions at the site of insertion, the insertion of additional sequences and even large-scale deletions or chromosomal rearrangements [88]. Genetic mapping and pollen viability analysis of 64 lines from the Salk T-DNA *Arabidopsis* mutant set showed that in 19% of the lines, a translocation had occurred (12 out of 64) [89]. The T-DNA may directly interrupt the expression of the gene where it inserted, but the translocation event itself can also alter the expression of genes [90]. Because of the uncertainty associated with the insertion site and number of copies of the gene of interest introduced into the host genome using the methods described above, it has long been the goal to target specific sites for the integration of new genes (gene targeting). The first successful gene targeting experiments in plants involved the rescue of an antibiotic resistance marker gene in *Nicotiana tabacum* [91]. Site-specific integration relies on homologous recombination, and efforts to exploit plant endogenous systems have focused on the use of site-specific nucleases, such as the induction of double-stranded break (DSB) sites. Early studies focused on the Cre/Lox system [92], induction of DSB sites using a rare restriction endonuclease [93] and, more recently, implementation of zinc-finger nucleases [94]. 

Plastid: Plastid transformation and expression of transgenes has several distinct advantages over *Agrobacterium*-mediated or biolistic nuclear transformation. Plastids are maternally inherited, and the risk of transgene escape through pollen is low. Plastid transformation methods rely upon integration of the gene of interest into the plastid genome through site-specific homologous recombination; thus, the precise site of integration is known, unlike *Agrobacterium*-mediated or biolistic transformation methods.

Perhaps the biggest advantage of plastid transformation and expression studies is that extremely high levels of transgene products can be achieved. Gene silencing does not occur in plastids, and there are multiple copies of the genome within each plastid and multiple plastids within each cell. Ruhlman *et al*. demonstrated that up to 72% of the total soluble leaf protein in transplastomic tobacco plants consisted of an engineered human proinsulin-cholera B toxin fusion [95].

To optimize the yield of a protein product, species-specific integration site sequences must be included in the transformation vector [96], coding sequences must be adjusted for the codon usage in the host organism [97,98] and specific N-terminal sequences can also stabilize the expressed protein [99,100]. The sequence of the plastid genome of the species that is to be transformed is used to generate cloning vectors that incorporate flanking sequences that are homologous to existing plastid sequences. The gene of interest is incorporated into the plastid genome via homologous recombination at a pre-determined site. In order to develop species-specific vectors, the plastid genome sequence needs to be known. The cotton chloroplast genomic sequence was used to develop probes to test the expression levels of 20 chloroplast genes in different cotton tissues under light- and dark-grown conditions. The results of this study can be used to specifically tailor cotton plastid transformation vectors for tissue and developmentally specific expression in plastids harbored in “light” and “dark” tissues [101].

The complete chloroplast genome has been determined for several crop plants (Table 2). Additional plant chloroplast genomic sequences are currently being determined at a rapid rate. Next generation sequencing methods are at the heart of these advances [102]. Modifications, including sequence enrichment, coupled with Illumina sequencing, such as those described by Stuhl *et al*., have the potential to facilitate rapid sequencing of multiple plastid genomes in a single lane [103]. As more species-specific chloroplast sequences come available, more plant species can be targeted for chloroplast engineering. However, the success of these studies will also depend upon optimization and the development of reproducible transformation, regeneration and selection protocols.

**Table 2:** Chloroplast genomes of crop plants.

Plant Chloroplast Genome	Date of Completion	Accession Number/Reference
Tobacco	April 27, 1998	Z00044
Rice (*japonica*)	March 29, 2001	X15901
Soybean	December 18, 2005	DQ317523
French Bean	August 7, 2007	DQ886273
Maize	April 18, 2005	X86563
Cotton (*G. hirsutum*)	May 3, 2006	DQ317523
*G. barbadense*	December 26, 2006	AP009123
*Gossypium* sp. (11 other species and races)	August 2, 2012	[104]

## 5. Conclusions

Cotton is important as a food and fiber crop and also a model system to study the interactions that occur when plants become polyploid. The majority of commercially grown cotton is *G. hirsutum*, an allotetraploid species. Because of the complexity and large size of the genome, the diploid ancestral species have been targeted for deep sequencing studies. The genome of *G. raimondii*, a New World species, has been completely sequenced, and efforts are ongoing to sequence an Old World representative. Determination of the genomes of the progenitors will facilitate the sequencing of the genome of *G. hirsutum* and lend insight into the fate of redundant genes that result from chromosome duplication events.

As the world’s increasing population continues to strain food supplies, producers are struggling to meet demands. This issue is further complicated by global climate change, pathogen infestations, use of potential food crops for biofuels, overuse of fertilizers and loss of soil due to cultivation practices. Alternate sources of food will be essential to meet this need. Genomic sequence information can be used to improve a variety of cotton traits that influence the nutritional quality of cottonseed protein, such as the reduction of gossypol and improved amino acid composition.

Development of cotton with other desirable properties, such as resistance to pathogens and drought tolerance, will be facilitated by knowledge of the ancestral diploid and commercially grown allotetraploid genomes. Ancestral strain germplasm may be a treasure trove of genetic information that will enable scientists and breeders to generate improved cotton varieties. Once the genes and pathways that are associated with the desired traits are identified, they need to be introduced or modified in commercially desirable varieties, either by conventional breeding or genetic engineering. Determination of the cotton nuclear and plastid genomes is central to the development of improved technology for engineering improved cotton. Plastid transformation has some obvious advantages over *Agrobacterium*-mediated nuclear transformation. Genomic information will facilitate the development of site-directed recombination strategies and reduce the undesirable aspects of current nuclear transformation protocols.
